# Transplantation of Human Menstrual Blood-Derived Mesenchymal Stem Cells Alleviates Alzheimer’s Disease-Like Pathology in APP/PS1 Transgenic Mice

**DOI:** 10.3389/fnmol.2018.00140

**Published:** 2018-04-24

**Authors:** Yongjia Zhao, Xin Chen, Yichen Wu, Yanling Wang, Yifei Li, Charlie Xiang

**Affiliations:** State Key Laboratory for Diagnosis and Treatment of Infectious Diseases, Collaborative Innovation Center for Diagnosis and Treatment of Infectious Diseases, The First Affiliated Hospital, College of Medicine, Zhejiang University, Hangzhou, China

**Keywords:** Alzheimer’s disease, human menstrual blood-derived mesenchymal stem, amyloid-β, microglia, tau

## Abstract

Extracellular β-amyloid (Aβ) plaques and neurofibrillary tangles (NFTs) are the pathological hallmarks of Alzheimer’s disease (AD). Mesenchymal stem cells (MSCs) have shown therapeutic efficacy in many neurodegenerative diseases, including AD. Human menstrual blood-derived stem cells (MenSCs) are a novel source of MSCs advantageous for their higher proliferation rate and because they are easy to obtain without ethical concerns. Although MenSCs have exhibited therapeutic efficacy in some diseases, their effects on AD remain elusive. In the present study, we showed that intracerebral transplantation of MenSCs dramatically improved the spatial learning and memory of APP/PS1 mice. In addition, MenSCs significantly ameliorated amyloid plaques and reduced tau hyperphosphorylation in APP/PS1 mice. Remarkably, we also found that intracerebral transplantation of MenSCs markedly increased several Aβ degrading enzymes and modulated a panel of proinflammatory cytokines associated with an altered microglial phenotype, suggesting an Aβ degrading and anti-inflammatory impact of MenSCs in the brains of APP/PS1 mice. In conclusion, these findings suggest that MenSCs are a promising therapeutic candidate for AD.

## Introduction

Alzheimer’s disease (AD) is the most prevalent type of dementia. AD is clinically characterized by progressive memory loss and cognitive dysfunction and neuropathologically characterized by extracellular deposition of amyloid beta (Aβ) plaques and intracellular neurofibrillary tangles (NTFs) composed of hyperphosphorylated tau proteins (Alzheimer’s Association, 2011; Ballard et al., 2011). Aβ is a proteolytic product of the transmembrane amyloid precursor protein (APP), and tau is a microtubule-associated protein that is enriched in neuronal axons and plays a pivotal role in axonal transport. In AD, extracellular accumulation of Aβ causes the formation of Aβ plaques, and tau becomes hyperphosphorylated, with consequent misfolding to further form NTFs. Several lines of evidence indicate that the deposition of Aβ aggravates phosphorylated tau and affects the surrounding central nervous system (CNS) resident cells, such as microglia, oligodendrocytes and neurons, ultimately causing neuroinflammation, neurodegeneration, and neuronal loss in AD pathologies (Götz et al., 2001; Cunningham, 2013; Zempel et al., 2013).

However, there are no effective therapies for AD involving pharmacological agents that target either Aβ or tau aggregation inhibitors (Doody et al., 2014; Salloway et al., 2014; Lovestone et al., 2015; Gauthier et al., 2016). Therefore, it is urgent to find alternative therapeutic strategies to treat AD. In recent years, with the development of stem cell technologies, mesenchymal stem cells (MSCs) with self-renewal, multipotency and cytokine secretion properties have been considered as a promising treatment for several neurodegenerative disorders, including AD. Several studies have revealed a beneficial effect of different types of MSCs on disease pathology and cognitive function in AD models. It has been reported that MSCs from bone marrow (BM-MSCs), umbilical cord (UC-MSCs) and adipose tissue (ASCs) can reduce Aβ deposition and tau hyperphosphorylation and improve impaired spatial memory, and the underlying mechanism of these beneficial effects has been suggested to be associated with microglia (Lee et al., 2010; Lee H.J. et al., 2012).

Human menstrual blood-derived stem cells (MenSCs) are isolated from the menstrual blood of women. MenSCs are a novel type of MSCs that are highly proliferative and multipotent; they exhibit beneficial phenotypes and properties and can also differentiate into the three germ lineages (Meng et al., 2007; Allickson and Xiang, 2012; Sugawara et al., 2014). Compared with other types of MSCs, MenSCs show higher proliferation rates and can be made readily to available through a safer and simpler route without inducing pain or being associated with ethical issues. Furthermore, MenSCs exhibit remarkable regenerative capacity and low immunogenicity (Khoury et al., 2014; Lai et al., 2015). These characteristics make MenSCs a potential therapeutic cell type for use in several disease models, such as type 1 diabetes, myocardial infarction, fulminant hepatic failure, acute lung injury and liver fibrosis (Wu et al., 2014; Zhang et al., 2013; Chen et al., 2017a,b; Xiang et al., 2017). Moreover, transplantation of human endometrial-derived stem cells was reported to restore dopamine production in a Parkinson’s disease model (Wolff et al., 2011). Thus, transplantation of MenSCs may be a potential therapeutic strategy for AD.

In the present study, we examined whether intracerebral transplantation of MenSCs could have beneficial effects on the neuropathology of AD in APP and presenilin one (PS1) double-transgenic mice. We discovered that MenSCs reduced Aβ deposition and tau hyperphosphorylation, improved cognitive decline, modulated microglia activation and restored Aβ clearance capacity in APP/PS1 transgenic mice.

## Materials and Methods

### Animals

Seven-month-old double-transgenic APPswe/PSEN1dE9 mice were used in this study. APPswe/PSEN1dE9 mice with a C57BL/6 background overexpress the mutant human genes APPswe (Swedish mutations K594N/M595L) and presenilin-1 with the exon-9 deletion (PS1-dE9). We used only male mice because of the gender-specific differences in the progression of AD pathology. APPswe/PSEN1dE9 mice and 7-month-old C57BL/6J mice were obtained from Nanjing BioMedical Research Institute of Nanjing University (Nanjing, China). Mice were housed under standard conditions including a 12-h light/dark cycle and allowed free access to food and water at the Laboratory Animal Center of Zhejiang University. All animal experiments were approved by the Animal Care and Use Committees of Zhejiang University.

### Cell Isolation and Culture

Human menstrual blood-derived stem cells were isolated and cultured as described previously (Meng et al., 2007; Wu et al., 2014). The volunteer donors provided informed consent, and the isolation procedure was approved by the Ethics Committee of the First Affiliated Hospital, College of Medicine, Zhejiang University, China. Briefly, menstrual blood samples were collected with a DivaCup (Kitchener, ON, Canada) from healthy women. Mononuclear cells were separated by density gradient centrifugation with Ficoll-Paque (Thermo Fisher Scientific, MA, United States). The interlayer cells were then collected and cultured in Chang medium (S-Evans Biosciences, Hangzhou, China). Cells were cultured in a tissue culture flask (Corning, Corning, NY, United States) at 37°C in a 5% CO_2_ humidified atmosphere. The medium was changed every 3 days and subcultured by using 0.25% trypsin EDTA (Thermo Fisher Scientific) until reaching approximately 70–80% confluence. The MenSCs used in the experiments were from passages 3 to 5.

### Characterization of the MenSCs

The surface markers of the MenSCs were evaluated using fluorescence-activated cell sorting. In brief, 1 × 10^6^ cells were collected and resuspended in staining buffer. MenSCs were then incubated with antibodies including CD29, CD34, CD45, CD73, CD90, CD105, CD117, human leukocyte antigen-DR (HLA-DR) and isotype control (BD, Franklin Lakes, NJ, United States) in the dark for 20 min. Stained cells were resuspended in staining buffer and analyzed with an FC 500 flow cytometer (Beckman, Brea, CA, United States).

### Transplantation of MenSCs in APP/PS1 Transgenic Mice

Human menstrual blood-derived stem cell suspensions or phosphate-buffered saline (PBS) alone were transplanted into APP/PS1 transgenic mice at the age of 7 months. Mice were anesthetized with isoflurane and fixed on a stereotaxic apparatus (RWD Life Science, Shenzhen, China). Using a 10-μl syringe (Hamilton) and an automated syringe pump (KD scientific, United States), 3 μl (approximately 1 × 10^5^ cells) of MenSC suspension was injected at a rate of 0.3 μl/min bilaterally into the hippocampus according to the following stereotaxic coordinates: 1.9 mm posterior to the bregma, 1.2 mm bilateral to the midline and 1.7 mm ventral to the skull surface. The syringe was kept in place for 5 min after the injection.

### Morris Water Maze

We used the Morris water maze to examine the spatial learning and memory function of APP/PS1 transgenic mice. Briefly, a white tank was filled with water to a depth of 20 cm. Opaque paint was added to the water before the tests. A circular platform (10 cm in diameter) was located at a fixed position approximately 1 cm below the water surface. One day before escape latency training, all mice were habituated to the maze. In escape latency training, the animals were subjected to four trials each day for six consecutive days. In each of the four trials, mice were placed in the water at four different starting positions equally spaced around the perimeter of the pool, and the mouse was given 60 s to swim to find the platform. If the mouse did not find the platform within 60 s, the mouse was guided to the platform and was allowed to remain on the platform for 10 s. The time the mice spent to find the platform was measured as the escape latency. On the 7th day, the platform was removed from the pool in the probe trial. Each animal was subjected to one trail and the number of times the mouse crossed the previous location of the platform in one minute was recorded.

### Tissue Preparation

Mice were anesthetized with 1% pentobarbital sodium after behavioral experiments and immediately transcardially perfused with cold saline and 4% paraformaldehyde fixative in PBS (pH 7.4). The brain was removed and post-fixed for 24 h at 4°C and then incubated consecutively in 20 and 30% sucrose solutions at 4°C. Sequential 30-μm coronal sections were prepared by using a cryostat (CryoStar NX50, Thermo Scientific) and stored at -20°C.

### Thioflavin S Staining

The brain sections were incubated with 1% thioflavin S solution dissolved in distilled water containing 50% ethanol for 5 min and differentiated in 50% ethanol three times. Fluorescence imaging was visualized by using an OLYMPUS IX83-FV3000-OSR (Olympus Corporation, Japan). To quantify the plaque load, the plaque areas of five sections of the cortex and hippocampus of the mice in each group were calculated and analyzed.

### Immunofluorescence

The brain sections were incubated for 1 h in PBS containing 10% normal goat serum and 0.1% Triton X-100 at room temperature. The brain sections were then incubated with primary antibodies overnight at 4°C. The following primary antibodies were used: mouse anti-6E10 monoclonal antibody (1:500; Covance, Princeton, NJ, United States) and rabbit anti-Iba-1 polyclonal antibody (1:500; Wako Chemicals, Japan). The brain sections were then rinsed in PBS and incubated with the following secondary antibodies: Alexa Fluo-488-conjugated goat anti-rabbit IgG antibody (1:500; Abcam) and Alexa Fluo-635-conjugated goat anti-mouse IgG antibody (1:500; Invitrogen). Finally, the brain sections were visualized using an OLYMPUS IX83-FV3000-OSR (Olympus Corporation, Japan).

### Western Blot Analysis

Hippocampal and cortical tissues were dissected and homogenized in RIPA buffer (Beyotime Biotechnology) supplemented with a cocktail of protease (Sigma) and phosphatase inhibitors (Thermo Fisher Scientific). The lysate was centrifuged at 12000 ×*g* for 15 min at 4°C. Protein concentration was determined using a bicinchoninic acid protein assay kit (Beyotime Biotechnology). Equal amounts of total protein (50 μg) in SDS sample buffer were subjected to 10% sodium dodecyl sulfate polyacrylamide gel electrophoresis (SDS-PAGE) and then electrophoretically transferred to immunoblotting polyvinylidene difluoride (PVDF) membranes. The membranes were treated with blocking solution (Thermo Fisher Scientific) at room temperature for 2 h, and then the membranes were incubated with primary antibodies overnight at 4°C. The following primary antibodies were used: mouse anti-APP polyclonal antibody (1:1000; Sigma), mouse anti-β-CTF polyclonal antibody (1:1000; Sigma), rabbit anti-BACE1 polyclonal antibody (1:1000; Abcam), mouse anti-NEP monoclonal antibody (1:1000; Abcam), rabbit anti-IDE polyclonal antibody (1:1000; Abcam), mouse anti-phospho-Tau (Ser202, Thr205) monoclonal antibody (AT8, 1:1000; Invitrogen), rabbit anti-phospho-tau (S396) monoclonal antibody (1:5000; Abcam), mouse anti-tau5 monoclonal antibody (1:1000; Abcam), rabbit anti-GSK-3β monoclonal antibody (1:1000; Cell Signaling Technology), rabbit anti-phospho-GSK3β monoclonal antibody (Ser9; 1:1000; Cell Signaling Technology), and mouse anti-beta actin monoclonal antibody (1:5000; Abcam). After washing in TBST, the membranes were incubated with goat anti-rabbit IgG (H+L)-HRP conjugate (1:3000; Bio-Rad) and goat anti-mouse IgG (H+L)-HRP conjugate (1:3000; Bio-Rad) for 1 h at room temperature. After washing again in TBST, immunoreactive bands were detected by chemiluminescence reagents (ECL, Bio-Rad). Images of the protein bands were acquired using a Tanon 4500 system. ImageJ was utilized to scan the pixel density of the protein bands of the resultant blots.

### Quantitative Real-Time PCR

Total RNA was extracted from hippocampal and cortical tissues using a total RNA kit (QIAGEN) according to the manufacturer’s instructions. Reverse transcription was performed using a PrimeScript^TM^ RT reagent kit (Takara), and real-time PCR was performed using a SYBR^®^ *Premix Ex Taq*^TM^ (Tli RNaseH Plus) kit (Takara) on an Applied Biosystems^®^ 7500 Fast Real-Time PCR Systems (Thermo Fisher Scientific). The ∆∆CT method was used to calculate the fold changes in gene expression level between target genes and GAPDH. The primers used in the experiments are listed in **Table 1**.

**Table 1:** Primers used for real-time PCR.

Gene	Forward (5′–3′)	Reverse (5′–3′)
IL-1β	GCCCATCCTCTGTGACTCAT	AGGCCACAGGTATTTTGTCG
TNF-α	CCACCACGCTCTTCTGTCTAC	TGGGCTACAGGCTTGTCACT
iNOS	ACCTTGTTCAGCTACGCCTT	CATTCCCAAATGTGCTTGTC
COX-2	ATGAGC ACAGGATTTGACCA	TGGGCTTCAGCAGTAATTTG
IL-6	ACTTCACAAGTCGGAGGCTT	TTGCCATTGCACAACTCTTT
IL-4	ATCATCGGCATTTTGAACGAGG	TGCAGCTCCATGAGAACACTA
Fizz1	CTGCTACTGGGTGTGCTTGT	GGCAGTTGCAAGTATCTCCA
YM1	TCTATGCCTTTGCTGGAATG	CAGGTCCAAACTTCCATCCT
Arg1	CTCCAAGCCAAAGTCCTTAGAG	GGAGCTGTCATTAGGGACATCA
NEP	CTCTCTGTGCTTGTCTTGCTC	GACGTTGCGTTTCAACCAGC
IDE	GAAGACAAACGGGAATACCGTG	CCGCTGAGGACTTGTCTGTG
GAPDH	CTCCACTCACGGCAAATTCA	GCCTCACCCCATTTGATGTT

### Aβ ELISA

Aβ ELISA was performed as previously described [23, 24] (Liu et al., 2014; Fang et al., 2016). Briefly, cortical and hippocampal tissues were homogenized in Tris-buffered saline (TBS) containing a protease inhibitor cocktail (Sigma) and then centrifuged at 16,000 ×*g* for 30 min at 4°C. The supernatant (TBS-soluble fraction) was collected, and the pellets were homogenized in TBS supplemented with 1% Triton X-100 (TBST) containing a protease inhibitor cocktail (Sigma), sonicated for 5 min at 4°C in a water bath, and then centrifuged at 16,000 ×*g* for another 30 min at 4°C. The supernatant (TBST-soluble fraction) was collected, and the pellets were extracted a third time with an ice-cold guanidine buffer (5 M guanidine HCl/50 mM Tris, pH 8.0) and referred to as the guanidine-soluble fraction. The protein concentration was determined using a bicinchoninic acid protein assay kit (Beyotime Biotechnology). The Aβ concentrations were detected by Aβ40 and Aβ42 ELISA kits (Invitrogen) following the manufacturer’s instructions. The levels of Aβ were normalized to the amount of total protein in the tissues.

### Statistical Analysis

Data are reported as the mean ± standard deviation (SD). Student’s *t*-test was used to compare the two groups. One-way analysis of variance (ANOVA) followed by *post hoc* Tukey’s test was used for multigroup comparisons. Statistical analyses were performed using GraphPad Prism 5 software (GraphPad Software). Values of *p* < 0.05 were considered statistically significant.

## Results

### Characterization of the MenSCs

**FIGURE 1:**
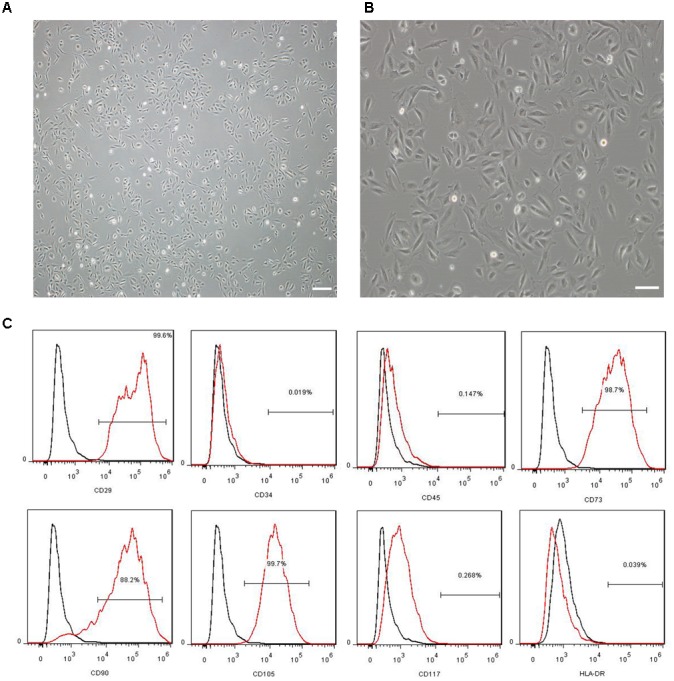
Characterization of MenSCs. **(A,B)** Representative images of MenSCs. Scale bars = 200 μm **(A)** and 100 μm **(B)**. **(C)** Flow cytometry analysis of the surface markers of MenSCs. The black lines represent the isotype control, and the red lines represent the level of surface markers. MenSCs, human menstrual blood-derived stem cells.

The morphology and immunophenotyping of MenSCs were similar to MSCs. As shown in **Figure 1**, MenSCs were spindle-shaped and plastic adherent cells (**Figure 1A**) that were positive for CD29, CD73, CD90, and CD105, which are markers of MSCs, and negative for CD34, CD117, and HLA-DR (**Figure 1B**). The results indicated that MenSCs were not haematopoietic and had low immunogenicity.

### MenSC Transplantation Ameliorates Spatial Learning and Memory Impairments in APP/PS1 Mice

**FIGURE 2:**
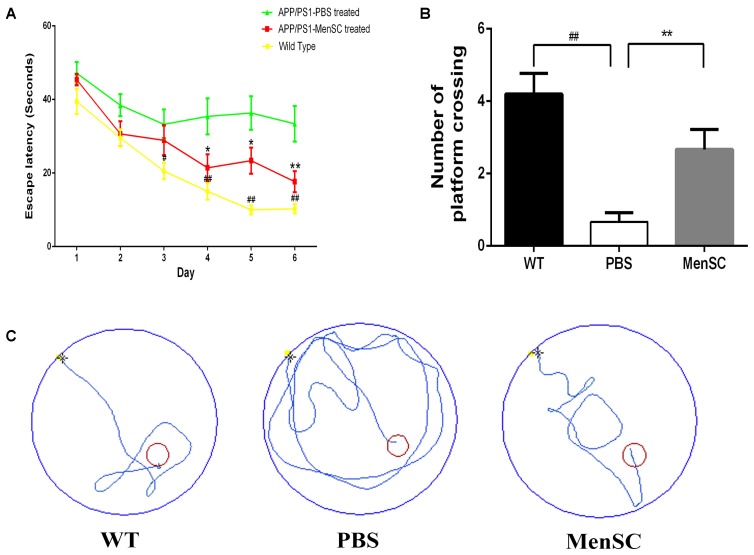
MenSC transplantation rescues spatial learning and memory impairments in APP/PS1 mice. **(A)** Escape latency to reach the hidden platform on 6 successive days in the Morris water maze for WT and APP/PS1 mice treated with MenSCs or PBS. For each day of training, the mean of 4 trials per day is presented. During training, MenSC-treated APP/PS1 mice required a shorter amount of time to reach the hidden platform than PBS-treated APP/PS1 mice. **(B)** The number of crossings of the location of the platform during the probe trial. The results represent the number of times each animal crossed the previous location of the platform within 1 min, which was removed in the probe trials. **(C)** Representative swimming paths of one mouse per group on day 6 illustrating the escape latency in the Morris water maze. The red circle shows the location of the former platform. Values are represented as the means ± SEM. Significant differences between PBS-treated and MenSC-treated APP/PS1 mice: ^∗^*p* < 0.05 and ^∗∗^*p* < 0.01, WT and PBS-treated APP/PS1 mice: ^#^*p* < 0.05 and ^##^*p* < 0.01, *n* = 15 per group. MenSCs, human menstrual blood-derived stem cells.

To estimate whether MenSC transplantation could improve cognition function and memory deficits of PBS-treated mice, MenSC-treated APP/PS1 mice and WT littermates, we used the Morris water maze to evaluate the spatial learning 21 days after MenSC transplantation. The Morris water maze is hippocampus dependent and can be used to evaluate the cognitive function of APP/PS1 mice. As presented in **Figure 2**, the PBS-treated APP/PS1 mice showed a significantly longer escape latency compared with their WT littermates, whereas the spatial learning and memory of APP/PS1 mice was rescued by treatment with MenSCs. APP/PS1 mice treated with MenSCs exhibited a significantly shorter escape latency than the mice treated with PBS (**Figure 2A**). Furthermore, the swimming paths of the WT and MenSC-treated APP/PS1 mice were target-oriented, while the PBS-treated APP/PS1 mice swam in circles to find the platform with no target orientation (**Figure 2C**). In the probe test, the platform was removed to evaluate the number of times each mouse crossed the original location of the platform in 60 s. The MenSC transplantation mice crossed the original location of the platform more often than the PBS-treated APP/PS1 mice (**Figure 2B**). These results indicated that MenSC transplantation could ameliorate learning and memory impairments in APP/PS1 mice.

### MenSC Transplantation Reduces Aβ Plaque Deposition and Levels in APP/PS1 Mice

**FIGURE 3:**
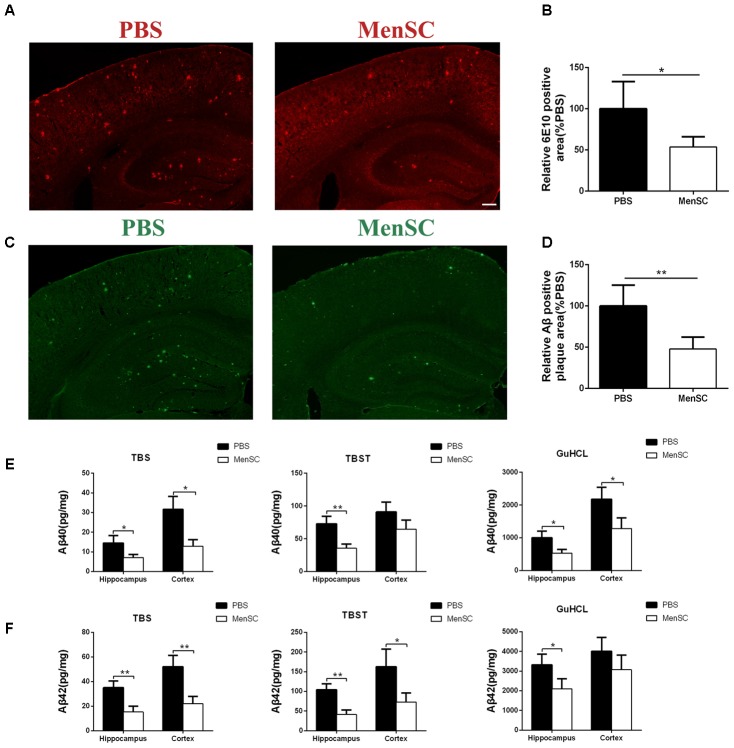
MenSC transplantation reduced Aβ deposition in APP/PS1 mice. **(A)** 6E10 antibody-stained brain sections of APP/PS1 mice treated with MenSCs compared with those of APP/PS1 mice treated with PBS. **(B)** Quantification of the 6E10-positive fraction showed a significant reduction in the hippocampus and the cortex of MenSC-treated APP/PS1 mice. Values are represented as a percentage of control (PBS-treated APP/PS1 mice). Scale bar = 200 μm, *n* = 5 per group. **(C)** Brain sections stained with thioflavin S in APP/PS1 mice treated with MenSCs or PBS. **(D)** Quantification of the thioflavin S-positive area demonstrated a marked decline in both the hippocampus and the cortex of APP/PS1 mice treated with MenSCs. Values are represented as a percentage of control (PBS-treated APP/PS1 mice). Scale bar = 200 μm, *n* = 5 per group. **(E,F)** Hippocampal and cortical tissues of APP/PS1 mice were homogenized and separated into TBS, TBST, and guanidine fractions. The levels of Aβ40 and Aβ42 were measured by using ELISA. Reductions in Aβ40 and Aβ42 were observed in the hippocampal and cortical samples of APP/PS1 mice treated with MenSCs. Values are represented as the means ± SD. Significant differences between PBS-treated and MenSC-treated APP/PS1 mice: ^∗^*p* < 0.05 and ^∗∗^*p* < 0.01, *n* = 3 per group. MenSCs, human menstrual blood-derived stem cells; Aβ, amyloid beta.

To assess the effects of MenSC transplantation on Aβ deposition, MenSCs were transplanted into the hippocampus of APP/PS1 mice at 7 months old, and the mice were subsequently sacrificed at 8 months old. The brain sections were stained with thioflavin S and 6E10 (Aβ antibody) to evaluate Aβ plaque deposition. Compared with PBS-treated mice, less Aβ plaques were observed in the hippocampus and the cortex of the MenSC-treated APP/PS1 mice as determined by thioflavin S staining (**Figures 3C,D**). Consistent with thioflavin S staining, quantitative analysis showed that 6E10-positive areas were significantly lower in the MenSC-transplanted APP/PS1 mice than in the PBS-treated mice (**Figures 3A,B**).

To further investigate the effect of MenSCs on Aβ pathology, the levels of different Aβ isoforms, Aβ40 and Aβ42, were measured in the hippocampal and cortical tissues by using ELISA. Aβ40 and Aβ42 are the most toxic Aβ isoforms and are enriched in monomers, oligomers and Aβ plaques in the hippocampus and cortex. The results revealed that MenSC administration markedly decreased the concentrations of Aβ40 and Aβ42 in TBS-, TBST-, and guanidine chloride-fractions in both the hippocampus and cortex in comparison with PBS administration (**Figures 3E,F**).

**FIGURE 4:**
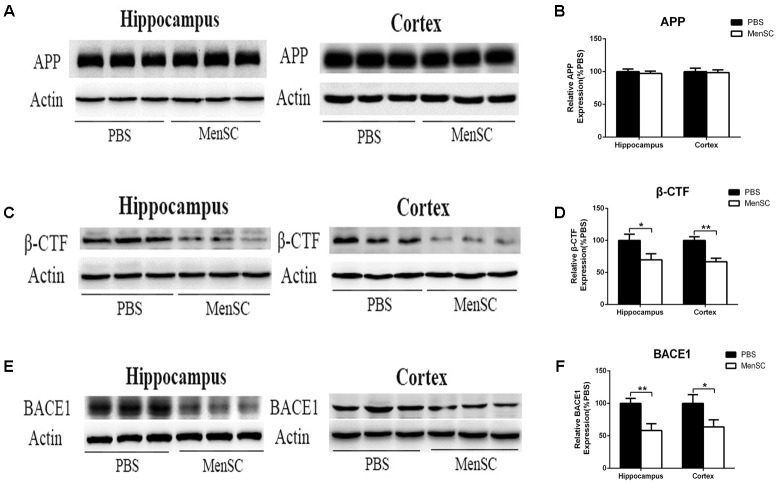
MenSC transplantation decreased BACE1 and β-CTF expression in the hippocampus and cortex of APP/PS1 mice. **(A,C,E)** Western blot analysis of APP, β-CTF, and BACE1 in hippocampal and cortical areas of APP/PS1 mice treated with MenSCs or PBS. **(B,D,F)** Quantification of Western blots in **(A,C,E)**, respectively, show protein as a percentage of control (PBS-treated APP/PS1 mice) normalized to actin. Values are represented as the means ± SD. Significant differences between PBS-treated and MenSC-treated APP/PS1 mice: ^∗^*p* < 0.05 and ^∗∗^*p* < 0.01, *n* = 3 per group. MenSCs, human menstrual blood-derived stem cells; APP, amyloid precursor protein.

Aβ is a proteolytic product of APP. To examine whether the reduction of Aβ deposition after MenSC transplantation was associated with the metabolic processing of APP, we used Western blot to investigate the expression of APP in the hippocampal and cortical tissues of APP/PS1 mice. Western blot analysis revealed that there were no significant differences in APP expression between APP/PS1 mice treated with MenSCs or PBS (**Figures 4A,B**), indicating that the level of APP expression was not changed after transplantation of MenSCs in the hippocampus and cortex of APP/PS1 mice. In the amyloidogenic pathway, APP is cleaved by β-secretase (BACE1) to generate a C-terminal fragment (β-CTF), and then β-CTF is further cleaved by γ-secretase to produce Aβ peptides. Therefore, we examined BACE1 by Western blot, and the results revealed that MenSC transplantation markedly decreased the levels of BACE1 in the brains of APP/PS1 mice (**Figures 4E,F**). Furthermore, western blot analysis of β-CTF suggested that β-CTF had declined in the MenSC-treated APP/PS1 mice (**Figures 4C,D**). Taken together, MenSC transplantation reduced Aβ deposition in the brains of APP/PS1 mice, probably by inhibiting the activity of β-secretase.

### MenSC Transplantation Stimulates Microglial Activation in the Brains of APP/PS1 Mice

**FIGURE 5:**
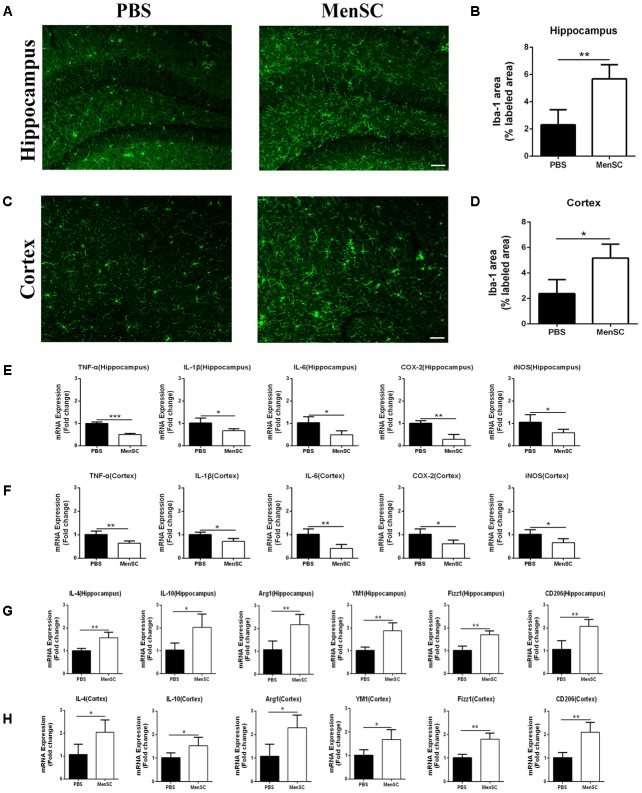
MenSC transplantation stimulated microglial activation and induced activated microglia toward the alternative phenotype. **(A,C)** Immunofluorescent staining for Iba-1 in brain sections of APP/PS1 mice treated with PBS or MenSCs. Scale bar = 100 μm, *n* = 5 per group. **(B,D)** Quantification of the percentages of the hippocampus and cortex exhibiting Iba-1 immunoreactivity. **(E,F)** Expression of TNF-α, IL-1β, IL-6, COX-2, and iNOS was measured by quantitative real-time PCR in the hippocampus and the cortex of APP/PS1 mice treated with PBS or MenSCs (*n* = 4 per group). The expression of these cytokines was significantly decreased in APP/PS1 mice after MenSC treatment. **(G,H)** The expression of IL-4, YM1, Arg1, CD206, IL-10, and Fizz1, which are markers of alternatively activated microglia, was measured by quantitative real-time PCR in the hippocampus and the cortex of APP/PS1 mice treated with PBS or MenSCs (*n* = 4 per group). There were significant increases in the mRNA expression of these markers in MenSC-treated APP/PS1 mice compared with APP/PS1 mice treated with PBS. Values are represented as the means ± SD. Significant differences between PBS-treated and MenSC-treated APP/PS1 mice: ^∗^*p* < 0.05 and ^∗∗^*p* < 0.01. MenSCs, human menstrual blood-derived stem cells.

In previous studies, our group proved that MenSCs could alleviate the inflammatory response in several diseases (Chen et al., 2017a; Xiang et al., 2017). Microglia are generally considered the immune cells in the CNS and are frequently associated with Aβ plaques in AD brains. Recent reports also have suggested that MSCs increase microglial activation in APP/PS1 mice (Lee et al., 2010; Lee H.J. et al., 2012). Therefore, we further evaluated whether MenSC transplantation could affect microglial activity in APP/PS1 mice. Mouse brain sections were stained with Iba-1, which is a marker of activated microglia. The results revealed that Iba-1-positive cells were widely distributed and markedly increased in the hippocampus and cortex of the MSC-treated APP/PS1 mice compared with the PBS-treated mice (**Figures 5A,C**). Quantitative analysis revealed that the MenSC-treated APP/PS1 mice displayed a greater area of Iba-1 immunoreactivity than the PBS-treated mice in both regions of the hippocampus and cortex (**Figures 5B,D**). Collectively, these results suggested that the activation of microglia could be improved after transplanting MenSCs into APP/PS1 mice.

### MenSC Transplantation Induces Activated Microglia Toward an Alternative Phenotype and Enhances Aβ-Degrading Enzyme Activity

**FIGURE 6:**
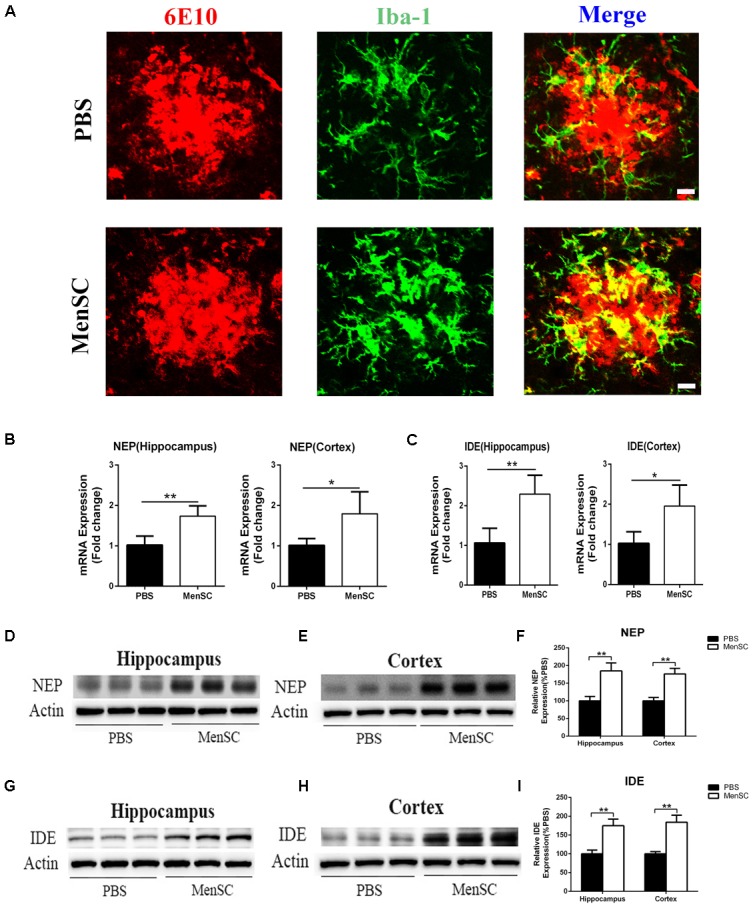
Transplanted MenSCs recruited microglia to cluster adjacent to the center of Aβ plaques and restored microglial Aβ clearance capacity in APP/PS1 mice. **(A)** Confocal microscopy was used to assess Iba-1 colocalization with 6E10 in APP/PS1 mice treated with MenSCs or PBS. Merged images of Iba-1 and 6E10 showed that more microglia clustered adjacent to the center of Aβ plaques in the brains of MenSC-treated APP/PS1 mice. Scale bar = 20 μm. **(B,C)** The expression of Aβ degrading enzymes NEP and IDE in the hippocampus and the cortex of APP/PS1 mice treated with PBS or MenSCs (*n* = 4). **(D,E,G,H)** Western blot analysis of NEP and IDE in hippocampal and cortical areas of APP/PS1 mice treated with MenSCs or PBS. **(F,I)** Western blots in **(D,E,G,H)**, respectively, show protein as a percentage of control (PBS-treated APP/PS1 mice) normalized to actin. Values are represented as the means ± SD. Significant differences between PBS-treated and MenSC-treated APP/PS1 mice: ^∗^*p* < 0.05 and ^∗∗^*p* < 0.01. MenSCs, human menstrual blood-derived stem cells; IDE, insulin-degrading enzyme; NEP, neprilysin (NEP).

Microglia are frequently related to Aβ plaques in AD brains. In previous experiments, we demonstrated that MenSC transplantation promoted the activation of microglia. It has also been reported that activated microglia can be either detrimental or beneficial in neuroinflammation. For example, microglia can be activated by Aβ deposition and increase the expression of proinflammatory cytokines, such as tumour necrosis factor (TNF)–α and interleukin (IL)-1β. Therefore, we quantified the mRNA expression of several proinflammatory factors and reactive oxygen species to investigate whether activated microglia aggravated neuroinflammation in APP/PS1 mice. Compared with the PBS-treated APP/PS1 mice, the expression of these cytokines was dramatically decreased in both the hippocampus and cortex of the MenSC-treated APP/PS1 mice. Interestingly, MenSCs administration stimulated microglial activation, yet the expression of proinflammatory cytokines decreased (**Figures 5E,F**). As microglial activation phenotypes are dynamic, we questioned whether MenSCs induced activated microglia to exhibit an alternative phenotype that exerts protective effects and reduces proinflammation cytokines to prevent neurotoxicity. IL-4, YM-1, Arg-1, Fizz1, and CD206 are associated with and strong expressed in alternative activation of microglia. Quantitative real-time PCR showed that the mRNA expression of IL-4, YM-1, Arg-1, Fizz1, and CD206 cytokines was markedly upregulated in MenSCs in both the hippocampus and the cortex of APP/PS1 mice (**Figures 5G,H**). Therefore, we considered that the activated microglia convert to an alternative phenotype, which is beneficial in reducing Aβ plaques. Next, colocalization of microglia with Aβ plaques in the brains of APP/PS1 mice was determined by immunofluorescence double staining of Iba-1 and 6E10 to further clarify the relationship between microglial activation and the reduction in Aβ deposition following transplantation of MenSCs. We noted that more Iba-1-positive cells were clustered adjacent to the center of Aβ plaques in MenSC-treated APP/PS1 mice, whereas Iba-1-positive cells tended to distribute around the Aβ plaques in PBS-treated APP/PS1 mice (**Figure 6A**). Alternatively activated microglia can secrete Aβ-degrading enzymes, including insulin-degrading enzyme (IDE) and neprilysin (NEP), which are two major proteases (Iwata et al., 2001; Farris et al., 2003). Therefore, we measured the mRNA expression of IDE and NEP in the hippocampus and cortex of APP/PS1 mice. The results showed that MenSC transplantation dramatically increased IDE and NEP mRNA expression in APP/PS1 mice (**Figures 6B,C**). Moreover, western blot analysis revealed that MenSC transplantation markedly increased the levels of NEP and IDE in the brains of APP/PS1 mice (**Figures 6D–I**). These results suggested that MenSC transplantation induced the activated microglia to display the alternative phenotype and enhanced Aβ-degrading enzyme activity in APP/PS1 mice, which both helped to reduce Aβ deposition.

### MenSC Transplantation Decreases Tau Hyperphosphorylation in APP/PS1 Mice

**FIGURE 7:**
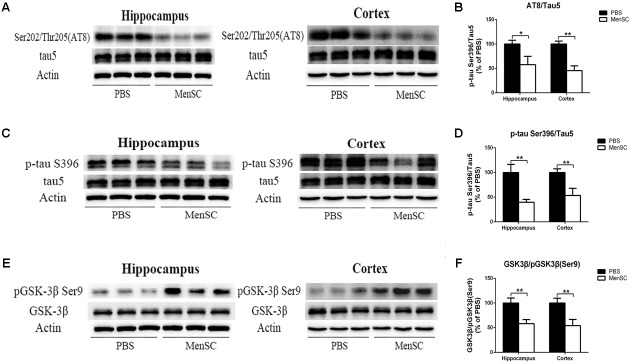
MenSC transplantation ameliorates tau hyperphosphorylation in APP/PS1 mice. **(A,C)** Western blot analysis of tau phosphorylation at Ser202/Thr205 (AT8) and Ser396 sites in the hippocampus and the cortex of APP/PS1 mice treated with PBS or MenSCs. **(B,D)** Quantification of the Western blot results in **(A,C)** presented as the ratio of AT8/Tau5 and Ser396/Tau5 as a percentage of control (PBS-treated APP/PS1 mice). **(E)** Western blot analysis of GSK3β and phosphorylated GSK3β at the Ser9 site in the hippocampus and the cortex of APP/PS1 mice treated with PBS or MenSCs. **(F)** Quantification of the Western blot results in **(E)** presented as the ratio of GSK3β/pGSK3β (Ser9). Values are represented as the means ± SD. Significant differences between PBS-treated and MenSC-treated APP/PS1 mice: ^∗^*p* < 0.05 and ^∗∗^*p* < 0.01, *n* = 3. MenSCs, human menstrual blood-derived stem cells; GSK3β, Glycogen synthase kinase 3 beta.

Neurofibrillary tangles composed of hyperphosphorylated tau protein are another pivotal pathological characteristic of AD. To assess whether MenSCs could affect tau pathology, tau phosphorylation at Ser202/Thr205 (AT8) and Ser396 sites was measured by Western blot. Western blot analysis showed that tau phosphorylation at Ser202/Thr205 (AT8) and Ser396 sites was dramatically downregulated in both the hippocampus and the cortex of MenSC-treated mice compared with PBS-treated mice, (**Figures 7A–D**). Next, to elucidate the mechanism through which MenSCs ameliorate tau phosphorylation in APP/PS1 mice, the levels of glycogen synthase kinase-3β (GSK-3β) were examined by using Western blot. GSK-3β is a serine/threonine protein kinase and is considered an important tau kinase. The expression of GSK-3β leads to tau phosphorylation at many sites, and GSK-3β phosphorylation at Ser9 leads to inhibition of its kinase activity (Llorens-Martín et al., 2014). Western blot analysis revealed that MenSC transplantation significantly increased the level of phosphorylated GSK-3β (Ser9) in the hippocampus and the cortex of APP/PS1 mice (**Figures 7E,F**). These results demonstrated that MenSC transplantation could reduce tau hyperphosphorylation by inactivating GSK-3β.

## Discussion

Human menstrual blood-derived stem cells isolated from menstrual fluids are a novel source of MSCs (**Figure 1**) and have important advantages such as the ability to perform repeated, simple sampling and to circumvent ethical issues compared with MSCs derived from other sources. Moreover, MenSCs can be periodically isolated from donors because the isolation procedure is non-invasive, thus ensuring higher therapeutic doses with fewer cell passages (Khoury et al., 2014; Alcayaga-Miranda et al., 2015). These characteristics make MenSCs an ideal candidate for AD therapy. In the current study, the effects of intracerebral MenSC transplantation on Aβ deposition, tau pathology and cognitive decline were evaluated in APP/PS1 double-transgenic mice for the first time. Our results show that MenSC transplantation reduced Aβ deposition (**Figure 3**), which was associated with an alleviation of abnormal APP processing (**Figure 4**) and restoration of Aβ clearance by microglia (**Figures 6B,C**), and decreased tau hyperphosphorylation, which was led to inactive GSK-3β (**Figure 7**), in APP/PS1 mice. Both the hippocampus and the cortex benefitted from MenSC transplantation. Concomitantly, MenSC transplantation stimulated microglial activation and induced activated microglia (**Figures 5A–D**) to express the alternative phenotype characterized by the secretion of anti-inflammatory cytokines (**Figures 5E–H**) rather than the neurotoxic phenotype. More importantly, MenSCs protected APP/PS1 mice against cognitive decline and memory deficits (**Figure 2**). Taken together, the treatment effectively attenuated AD pathology by targeting multiple key pathways in APP/PS1 mice.

Analysis of 6E10 and thioflavin S staining revealed that Aβ deposition in the hippocampus and cortex of MenSC-treated APP/PS1 mice was significantly reduced (**Figures 3A–D**), and the ELISA results for Aβ40 and Aβ42 were consistent with 6E10 and thioflavin S staining (**Figures 3E,F**). In the amyloidogenic pathway, APP is cleaved by β-secretase (BACE1) to generate a C-terminal fragment (β-CTF), and then β-CTF is further cleaved by γ-secretase to produce Aβ peptides (Zhang et al., 2011). During this process, BACE1 is considered the initial and rate-limiting enzyme (Ye and Cai, 2014). Therefore, many studies have focused on elucidating the role of BACE1 in AD. It has been reported that BACE1 knockout (BACE1^-/-^) prevents the development of AD pathologies and cognitive decline in mouse models of AD (Ohno et al., 2004). Moreover, BACE1 activity increases with age and is increased in the brains of AD patients (Yang et al., 2003; Fukumoto et al., 2004). Here, we observed that MenSC transplantation decreased BACE1 and β-CTF levels in the hippocampus and cortex of APP/PS1 mice (**Figure 4**). According to our results, we attribute the beneficial effect of MenSCs on reducing Aβ deposition, at least in part, to inhibiting BACE1 activity.

Microglia, the resident immune cells in the CNS, play a critical role in immune surveillance of the brain (Boche et al., 2013). Activated microglia exert both toxic and beneficial effects depending on their phenotype in AD progression. It is well established that Aβ plaques sustain the activation of microglia, resulting in constant production of inflammatory cytokines and reactive oxygen species, which lead to neurodegeneration (Pimplikar, 2014; Ransohoff, 2016). On the other hand, alternatively activated microglia exert neuroprotective effects in AD that depend on attenuating inflammation and phagocytizing Aβ, which are related to the increased secretion of neurotrophic factors and proteases (Heneka et al., 2014). Our current findings showed that MenSC transplantation stimulated microglial activation (**Figures 5A–D**). Next, real-time PCR was performed to examine the expression of IL-1β and TNF-α to investigate whether activated microglia stimulated by MenSCs produced proinflammatory cytokines. Strikingly, we observed that the expression of these proinflammatory factors was significantly decreased after MenSC transplantation in the brains of APP/PS1 mice (**Figures 5E,F**). It has been reported that MSC transplantation can induce activated microglia to express the alternative phenotype rather than the neurotoxic phenotype in APP/PS1 mice (Lee et al., 2010; Lee H.J. et al., 2012). Thus, we investigated whether MenSC transplantation induced activated microglia to display the alternative phenotype. It is widely acknowledged that IL-4 is a pivotal anti-inflammatory cytokine that enhances the alternative phenotype of activated microglia *in vivo* and improves Aβ removal (Heneka et al., 2014; Latta et al., 2015). YM1, Arg1, Fizz1 and CD206 which are recognized as markers of the alternative activation of macrophages, are also strongly expressed in alternatively activated microglia. It is compelling that MenSC transplantation dramatically increased the mRNA levels of IL-4, YM1, Arg1, Fizz1, and CD206 in both the hippocampus and the cortex of APP/PS1 mice (**Figures 5G,H**). These results clearly demonstrated that MenSC transplantation induced activated microglia toward the alternative phenotype, which exerted beneficial effects in AD.

Insulin-degrading enzyme and neprilysin are the major Aβ-degrading enzymes and can be secreted by microglia in the brain (Tuppo and Arias, 2005). However, impaired and dysfunctional microglia decrease the capacity of Aβ clearance in AD model mice due to reduced IDE and NEP secretion (Hickman et al., 2008). Recent studies have revealed that soluble intracellular adhesion molecule-1 (sICAM-1) secreted by human umbilical cord blood-derived MSCs decreased Aβ plaques by inducing NEP expression in microglia in APP/PS1 mice (Kim et al., 2012), and soluble CCL5 derived from bone marrow-derived MSCs modulated the microglial activation status and reduced Aβ deposition by expression of Aβ-degrading enzymes in APP/PS1 mice (Lee J.K. et al., 2012). Moreover, it has been reported that MSC transplantation can promote the alternative activation of microglia and enhance the secretion of NEP and IDE by alternatively activated microglia (Ma et al., 2013). In our study, we observed that activated microglia clustered adjacent to the center of Aβ plaques in response to MenSC transplantation, whereas activated microglia distributed around the Aβ plaques in PBS-treated mice (**Figure 6A**). The quantitative real-time PCR and western blot analysis showed that MenSC transplantation significantly increased the expression of NEP and IDE in the hippocampus and the cortex of APP/PS1 mice (**Figures 6B–I**). Based on these results, we proposed that MenSC transplantation induced activated microglia to convert to the alternative phenotype and that the positive effects of MenSCs were conferred through upregulated levels of Aβ-degrading enzymes, leading to increased Aβ clearance. The beneficial effect of MenSC transplantation on reducing Aβ deposition was associated with inhibiting BACE1 activity and restoring Aβ-clearance via microglia. In addition to these two pathways, the influx and efflux of brain Aβ also mediate Aβ clearance, which may be another pathway that could improve Aβ clearance and deserves further study.

Intracellular NFTs composed of phosphorylated microtubule-associated tau protein is another pathological hallmark of AD (Ballard et al., 2011). Under abnormal phosphorylation, tau lessens its affinity and separates from microtubules, which damages microtubule function and affects axonal transport (Spillantini and Goedert, 2013). Among the main aberrantly hyperphosphorylated sites on tau, Ser202/Thr205 (AT8) and Ser396 are the pathological phosphorylated sites (Plattner et al., 2006). It has been reported that increased phosphorylation of the Ser202/Thr205 (AT8) site decreased mitochondrial transport in axons in AD, leading to axonal degeneration (Shahpasand et al., 2012). Recent study has shown that tau phosphorylation at Ser 396 is markedly increased in hippocampus of 5 × FAD mice compared with wild type mice. It occurs earlier than the appearance of learning and memory disorders (Kanno et al., 2014). Moreover, phosphorylated tau subsequently aggregates to form paired helical filaments (PHFs) and further forms NFTs, which lead to neuronal dysfunction and loss. The density of NFTs is directly correlated with disease severity and the degree of dementia in AD patients (Alonso et al., 1996; Ballatore et al., 2007). Therefore, phosphorylated tau has become a novel therapeutic target in AD. In the current study, we observed that MenSC transplantation dramatically reduced tau phosphorylation at Ser202/Thr205 (AT8) and Ser396 sites in the brains of APP/PS1 mice (**Figures 7A–D**). These data strongly demonstrate that MenSC transplantation can inhibit tau phosphorylation in APP/PS1 mice.

Several kinases have been reported to phosphorylate tau. Glycogen synthase kinase-3β (GSK3β) is one of the most important kinases due to its ability to phosphorylate tau at the majority of its serine/threonine sites, which cause associated toxicities in AD (Medina et al., 2011; Llorens-Martín et al., 2014). GSK3β leads to tau hyperphosphorylation at many sites *in vitro* (Rankin et al., 2007) and *in vivo* (Liu et al., 2003). Recent studies have been reported that Ser396 is a major substrate for GSK-3β (Liu et al., 2007) and Ser202/Thr205 (AT8) is one of the moderate phosphorylation sites for GSK-3β (Illenberger et al., 1998). Researchers also have found that several small molecule inhibitors of GSK-3β could reduce tau phosphorylation at Ser396 *in vivo* (Selenica et al., 2007). Overexpression of GSK-3β in transgenic mice leads to tau phosphorylation (Engel et al., 2006), and GSK-3β inhibitors significantly alleviate tau phosphorylation and tau-induced neurodegeneration (Noble et al., 2005). The activity of GSK-3β is regulated by autophosphorylation at Ser9, which results in a kinase-inactive state (Medina et al., 2011). In the previous study, our group demonstrated that MenSCs could inactivate GSK-3β in order to promote the repair of damaged lung tissue in acute lung injury (Xiang et al., 2017). Therefore, GSK-3β was selected in our study. We found that MenSC transplantation markedly upregulated the phosphorylation of GSK-3β at Ser9, which resulted in GSK-3β inactivity in both the hippocampus and the cortex of APP/PS1 mice (**Figures 7E,F**). These results indicate that MenSC transplantation could alleviate tau phosphorylation in the brains of APP/PS1 mice, which is associated with the suppression of GSK-3β activity. Moreover, GSK-3β is reported not only to phosphorylate tau, but also to regulate the production of Aβ. GSK-3β could regulate β-secretase (BACE1) expression to control Aβ formation. In the amyloidogenic pathway, APP is cleaved by β-secretase (BACE1) to generate β-CTF. Subsequently, γ-secretase is cleaved β-CTF to release Aβ, which tends to aggregate to form senile plaques. It has been reported that GSK-3β inhibition reduced BACE1-mediated cleavage of APP through NF-κB signal pathway in the APP/PS1 transgenic mouse model (Ly et al., 2013), further supporting that the inhibition of GSK-3β reduces Aβ pathology. Our results have shown that MenSCs transplantation reduced BACE1 expression and β-CTF in APP/PS1 mice (**Figure 4**), which is possibly related to inactivate GSK-3β. Accumulating evidence suggest that GSK-3β affects inflammatory. Several pro-inflammatory cytokines such as TNF have been found to activate GSK-3β, whereas GSK-3β inhibitors could protect from inflammatory conditions in animal models (Wyss-Coray and Rogers, 2012). IL-1β has been shown to increase tau phosphorylation through increasing GSK-3β and MAPK (Ghosh et al., 2013). These evidence demonstrate that GSK-3β plays a pivotal and central role in the pathogenesis of AD.

Excessive Aβ accumulation and hyperphosphorylated tau are associated with cognitive decline and memory deficits in AD model mice (Chen et al., 2000). The extent to which MenSC transplantation can attenuate cognitive impairment was studied here using the Morris water maze. In our study, MenSC transplantation resulted in improved spatial learning of APP/PS1 mice. Furthermore, the WT and MenSC-treated APP/PS1 mice showed a target-oriented swimming pattern, while the PBS-treated APP/PS1 mice swam in circles. These results suggest that the PBS-treated APP/PS1 mice reached the platform randomly and demonstrate that MenSC treatment dramatically attenuated cognitive decline of APP/PS1 mice.

## Conclusion

In conclusion, our results show that MenSC transplantation can ameliorate AD pathology and cognitive deficits in a transgenic mouse model of AD. However, the mechanisms of the MenSC-mediated improvements in APP/PS1 mice remain obscure. Most recent studies have considered that the neuroprotective effects of MSC transplantation are mainly stimulated by the paracrine effect (Drago et al., 2013). Our group also demonstrated that the beneficial effects of MenSC transplantation in mice with liver fibrosis and type 1 diabetes are mainly promoted by paracrine effects (Wu et al., 2014; Chen et al., 2017b). More research will be aimed at elaborating the exact paracrine effects of MenSCs and determining which factors secreted by MenSCs are capable of alleviating AD pathology and inducing activated microglia phenotype turn-over toward neuroprotection.

Here, we show that intracerebral MenSC transplantation reduces Aβ deposition mediated by BACE1 activity and Aβ-degrading enzymes, attenuates tau phosphorylation mediated by GSK3β, induces activated microglia phenotype turn-over toward neuroprotection and improves cognitive decline in APP/PS1 mice. These data indicate that MenSCs may be a novel cell therapy for AD.

## Author Contributions

YZ and CX designed the research work. YZ performed the intracerebral transplantation of MenSCs. YZ, XC, and YiW performed the Morris water maze analyses and biochemical analyses. YZ, YaW, and YL performed the RT-PCR analyses and histopathological analyses. YZ and XC interpreted the data and drafted the manuscript. All authors read and approved the final manuscript.

## Conflict of Interest Statement

The authors declare that the research was conducted in the absence of any commercial or financial relationships that could be construed as a potential conflict of interest.
